# Differences in inhibitory control between Progressive Supranuclear Palsy and Parkinson's Disease

**DOI:** 10.1002/alz70857_102240

**Published:** 2025-12-24

**Authors:** Federico Castano, Carrillo Juan, Maria Roca, Oscar Gershanik, Anabel Chade, Gonzalo Gomez‐Arevalo, Blas Couto

**Affiliations:** ^1^ Instituto de Neurología Cognitiva (INECO), CABA, Buenos Aires, Argentina; ^2^ Laboratory of Progressive Neuromotor and Neurobehavioral Disorders at Instituto de Neurociencia Cognitiva y Traslacional (INCYT), CABA, Buenos Aires, Argentina; ^3^ Laboratory of Language and Neuropsychology at Instituto de Neurociencia Cognitiva y Traslacional (INCYT), CABA, Buenos Aires, Argentina; ^4^ Neurologia, Universidad Favaloro, CABA, Buenos Aires, Argentina; ^5^ Unidad de Movimientos Anormales, Institute of Neuroscience of the Fundacion Favaloro, CABA, Buenos Aires, Argentina

## Abstract

**Background:**

Progressive Supranuclear Palsy (PSP) shares cognitive and behavioral changes with Parkinson's Disease (PD) that challenge their differential diagnosis, especially in early stages. Changes in inhibitory control associated with impulse control disorders are often seen in PD, and related to dopaminergic treatment. We aimed to uncover distinct neuropsychological profiles particularly in the amount and type of errors within a standard cognitive assessment protocol.

**Method:**

Participants included 29 patients with diagnosis of clinically probable PSP and 41 early‐PD patients matched by disease duration. Assessment consisted of a standard 90‐minute battery of neuropsychology widely‐validated tests. We analyzed the presence of repetitions and perseverative errors across several tests: phonological and semantic fluency, Trail‐making tests A/B, verbal memory lists (immediate and delayed recall), Go/No‐Go, and Wisconsin‐card sorting test. Results were co‐variated by an attentional score.

**Result:**

PSP patients exhibited significantly poorer performance than PD in verbal fluency, attention, and processing speed (TMT‐A/B). In addition, PSP showed higher frequencies of perseverative and commission errors highlighting a specific profile with decreased flexibility and impaired inhibitory control in PSP compared to early‐PD. PD patients did not use higher doses of dopaminergic treatment than PSP.

**Conclusion:**

The findings underscore the diagnostic value of specific error patterns in routine tasks used for assessing cognitive profiles in neurology. These profiles could serve as a bed‐side tool to help in the early differentiation of PSP and PD, and show valuable insights into the pathophysiological mechanisms of inhibitory control in both diseases.

